# What Are the Precursor and Early Lesions of Peripheral Intrahepatic Cholangiocarcinoma?

**DOI:** 10.1155/2014/805973

**Published:** 2014-04-22

**Authors:** Yasuni Nakanuma, Akemi Tsutsui, Xiang Shan Ren, Kenichi Harada, Yasunori Sato, Motoko Sasaki

**Affiliations:** ^1^Department of Human Pathology, Kanazawa University Graduate School of Medicine, Kanazawa 920-8640, Japan; ^2^Department of Pathology, Shizuoka Cancer Center, Shizuoka 411-8777, Japan

## Abstract

Cholangiocarcinoma (CC) is divided into distal, perihilar, and intrahepatic CCs (ICCS), and are further subdivided into large bile duct ICC and peripheral ICC. In distal and perihilar CC and large duct ICC, biliary intraepithelial neoplasm (BilIN) and intraductal papillary neoplasm (IPN) have been proposed as precursor lesions. Peripheral ICC, bile duct adenoma (BDA), biliary adenofibroma (BAF), and von Meyenburg complexes (VMCs) are reportedly followed by development of ICCs. Herein, we surveyed these candidate precursor lesions in the background liver of 37 cases of peripheral ICC and controls (perihilar CC, 34 cases; hepatocellular carcinoma, 34 cases and combined hepatocellular cholangiocarcinoma, 25 cases). In the background liver of peripheral ICC, BDA and BAF were not found, but there were not infrequently foci of BDA-like lesions and atypical bile duct lesions involving small bile ducts (32.4% and 10.8%, resp.). VMCs were equally found in peripheral CCs and also control CCs. In conclusion, BDA, BAF, and VMCs are a possible precursor lesion of a minority of peripheral CCs, and BDA-like lesions and atypical bile duct lesions involving small bile ducts may also be related to the development of peripheral ICC. Further pathologic studies on these lesions are warranted for analysis of development of peripheral ICCs.

## 1. Introduction

Cholangiocarcinoma (CC) is an intractable malignant tumor with a poor prognosis. Surgical resection of CC at an early stage is crucial to improve the prognosis of CC patients [1, 2]. However, this procedure is not applicable to the majority of these patients because most CCs are diagnosed or detected at an advanced stage, and treatment options remain limited [3]. CC is generally divided into distal and perihilar CCs and intrahepatic CC (ICC) [4, 5], and their clinicopathological features, risk factors, and epidemiology are different among them [2, 6, 7].

ICC is the second most common liver primary tumor after HCC, and its incidence has increased in recent years [7–9]. ICCs themselves are known to exhibit heterogeneity in their location in the liver, histopathologies, and expression of markers [8], and this heterogeneity may reflect the heterogeneous cholangiocytes along the biliary tree [4, 6, 10, 11]. ICC can be further divided into large bile duct ICC and peripheral ICC [12]. In distal, perihilar, and intrahepatic large duct, cylindrical mucin-producing cholangiocytes are located in large bile ducts, while cuboidal non-mucin-producing cholangiocytes are located in small bile ducts and bile ductules containing bipotential hepatic progenitor cells (HPCs). Among the ICCs, peripheral ICCs, which typically present as a mass or nodular lesion, are currently considered to be derived from the cells lining small bile ducts, and large bile duct ICCs resemble perihilar CC. Many studies have recently suggested that stem or progenitor cells located in the periportal area play a role in neoplastic and nonneoplastic hepatobiliary lesions in adults [6, 13]. Liver cirrhosis, particularly that associated with HBV and HCV, has been reported as a risk factor for peripheral ICC, and hepatic stem/progenitor cells located at the Canals of Hering may be involved in its carcinogenesis in such cases [2, 7, 14].

Precursor and early neoplastic lesions of CCs may provide an insight into identifying a more likely candidate for precursor lesions preceding the development of CCs. For example, two types of preneoplastic or early neoplastic lesions were recently identified for large duct ICC, perihilar and distal CCs, and gallbladder carcinomas: biliary intraepithelial neoplasm (BilIN) and intraductal papillary neoplasm (IPN) [4, 5, 15]. The former is a flat and microscopically recognizable lesion that is categorized into three grades: BilIN-1, -2, and -3, while the latter is a grossly visible papillary lesion in the dilated bile duct that is graded as low-intermediate and high. While many clinical and molecular studies have examined peripheral ICC [2, 7, 16], the precursor and premalignant lesions defined pathologically and histogenesis of peripheral CC have yet to be determined in detail.

So far, several candidate lesions, possibly followed by or associated with the development of peripheral ICC, have been proposed: bile duct adenoma, biliary adenofibroma, and von Meyenburg complexes (VMCs) [15, 17, 18]. In addition, low grade biliary epithelial malignancies such as those with ductal plate malformation- (DPM-) like features and cholangiolocellular carcinoma (bile ductular carcinoma) are known to contain the foci of classical or conventional CC [13, 18], suggesting their progression from low grade biliary epithelial malignancies to conventional CC with more malignant biological behaviors [13, 19].

Herein, we, first, reviewed the pathological features of the above-mentioned, several candidate lesions possibly followed by peripheral ICC, in the literatures. Second, we histologically surveyed such candidate lesions in the background liver of peripheral ICC in comparison with perihilar CC, HCC, and combined hepatocellular-cholangiocarcinoma (cHCC-CC) by using a total of 114 surgically resected cases. These cases were collected from the file of hepatobiliary diseases in the Department of Pathology and affiliated hospitals, and their main clinicopathological features are shown in Table 1. Finally, we discussed low grade or borderline biliary malignancies frequently followed by ordinary peripheral ICC with more aggressive behaviors.

## 2. Precursor and Premalignant Lesions of Peripheral ICC

New insights into the molecular and genetic mechanisms contributing to the pathogenesis of peripheral ICCs are emerging from recent epidemiological, genome-wide profiling, and laboratory-based studies [2, 7, 16]; however, their exact signaling pathways and etiology have yet to be elucidated in detail. For further elucidation and clarification of these mechanisms, a pathological entity of precursor or early neoplastic lesions in peripheral ICC cases needs to be addressed. The detailed analysis of these candidate precursors or premalignant biliary epithelial lesions may also assist in the search for the molecular and genetic studies on peripheral ICC. While the precursor lesions of peripheral ICC have not yet been identified, it appears reasonable to assume that its carcinogenesis may involve small intrahepatic bile ducts such as bile ductules, small interlobular bile ducts, or hepatic stem/progenitor cells including the canals of Hering, as indicated by the location of peripheral ICC in the liver [7, 14, 20, 21]. So far, the following lesions are known to be occasionally followed by the development of peripheral ICC in the literatures: bile duct adenoma, VMCs, and biliary adenofibroma [15, 17, 18]. While the incidence of malignant transformation may be quite low in these benign tumors, the pathological clarification and categorization of such lesions as well as more detailed molecular studies on these lesions may lead to better understanding of the carcinogenesis of peripheral ICC and may develop novel therapeutic strategies against intractable ICC.

### 2.1. Pathology of Candidate Precursor and Premalignant Lesions

**Figure 1:**
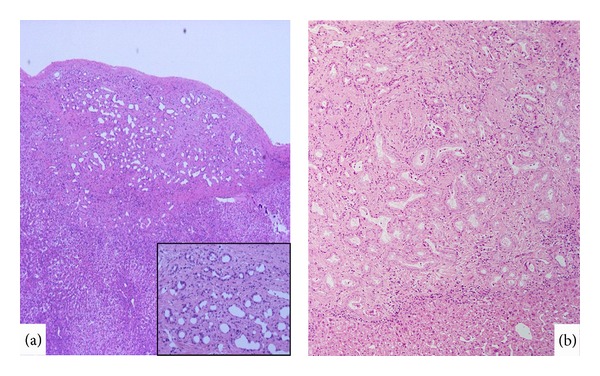
Bile duct adenoma. (a) Ordinary bile duct adenoma is seen under hepatic capsule, and it is composed of well-developed bile ducts of interlobular bile duct sizes (inset). HE. (b) Occasionally, bile duct adenoma with columnar epithelium and plenty cytoplasm shows compact growth. HE.

*Bile duct adenoma* (BDA), which is also called peribiliary gland hamartoma, is regarded as a benign tumor or tumorous lesion composed of many small, uniformly sized ducts with cuboidal cells (bile ductular component) resembling bile ductular reactions (DRs) and variable fibrous stroma [18, 22, 23]. Allaire et al. reviewed the morphological spectrum of BDA using 152 cases [18] and found that all BDAs were asymptomatic nodules discovered incidentally during intra-abdominal surgery or at autopsy. BDAs are typically single, ranged in size from 1 to 20 mm, usually subcapsular, and well-circumscribed but nonencapsulated. BDA is known to be histologically composed of benign, noncystic ductules or interlobular bile ducts and variable degrees of inflammation and fibrosis (Figure 1(a)). Mucin is frequently detected in the cytoplasm of BDA. The immunophenotype of these ductules was shown to be similar to that of interlobular bile ducts. BDA could be distinguished from adenocarcinoma by the absence, in the former, of nuclear hyperchromasia, mitotic activity, and vascular invasion.

The biological nature of BDA has not yet been clarified in detail; however, it is generally regarded as a reactive process to a focal injury. However, interestingly, some BDAs are known to exhibit neoplastic potential followed by classical peripheral ICC [18, 22]. It seems therefore plausible that BDAs may be a heterogeneous group or have a broad morphological spectrum. Particularly, BDAs arising in chronic advanced liver diseases such as chronic viral hepatitis, those found deep in the liver parenchyma, and those with some biliary epithelial atypia and unusual features but not enough to be diagnosed as a malignancy (Figure 1(b)) may be precursor lesions for peripheral CC.

**Figure 2:**
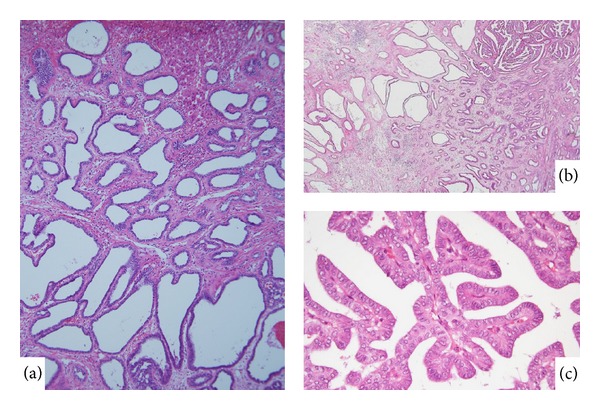
Biliary adenofibroma. (a) Tubular structures with fibrous stroma characterize this tumor. Focally, micropapillary features are found in the left lower corner. (b) Papillary features with complicated structures suggest an imminent malignant transformation. HE.

*Biliary adenofibroma *is characterized by a complex tubulocystic biliary epithelial tumor and abundant fibroblastic stromal components [24]. The lining epithelia of these complexes are columnar or cuboidal epithelia positive for the biliary cytokeratin marker and negative for mucin staining (Figure 2(a)) [24]. While the tumor is similar to VMCs, the large size of the lesion and absence of any typical MC characterize biliany adenofibroma. While most biliary adenofibromas are benign, one case of biliary adenofibroma with malignant transformation, recurrence, and metastasis was reported previously [17]. The patient was diagnosed with biliary adenofibroma with malignant epithelial transformation following a pathological examination of the resected specimen. He discontinued the follow-up program for 1 year but was then admitted to the hospital with abdominal enlargement and right upper quadrant pain. A needle biopsy was performed, and a pathological examination of the biopsy specimen confirmed the recurrence of malignant biliary adenofibroma.

We recently encountered one case (69 years, female) of biliary adenofibroma with imminent malignant transformation (Tsutsui A, personal communication). The tumor (3.5 cm in a diameter) was located under the hepatic capsule in the left hepatic lobe. Most of the lesion was compatible with biliary adenofibroma, but some of it had a papillary configuration and more dysplasia (Figures 2(b) and 2(c)). A number of unusual features suggesting neoplasm were focally present, including intraluminal bile concretions and apocrine-like epithelial changes. While its expansile growth, presence of mitoses, the foci of epithelial tufting, and cellular atypia favor a neoplastic process, features indicative of overt malignancy and invasion or metastasis were not found. This case suggests that while biliary fibroadenoma is a benign tumor, it is possibly followed by the malignant transformation.

**Figure 3:**
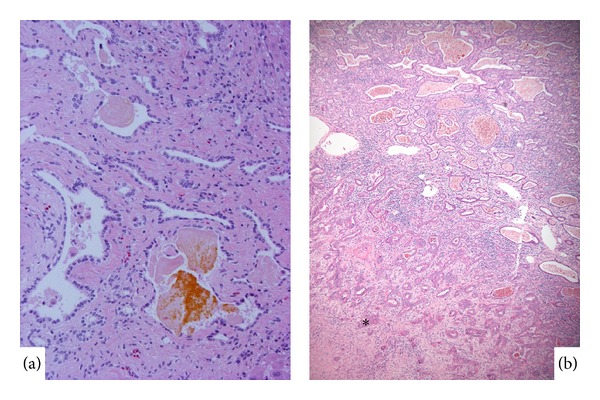
(a) Von Meyenburg complex showing a variable luminal dilatation and condensed bile. (b) Upper part shows low grade intrahepatic cholangiocarcinoma with features of von Meyenburg complex, and there are foci of ordinary cholangiocarcinoma in the low half (∗). HE.

*VMCs* which are also called biliary microhamartoma are histopathological lesions composed of irregular small bile duct or dilated ductular structures, frequently containing concentrated bile, with a fibrous stroma, and are found at the interface of portal tracts (Figure 3(a)) [25]. While this lesion is generally considered to be a congenital or hamartomatous lesion, it is not typically found in infants and is not infrequently found in adult livers, suggesting the participation of acquired factors in the development of VMCs [26]. Several lesions are commonly found in the same liver and are occasionally multiple. Whereas VMCs are generally regarded as benign, recent case reports of CC arising from VMCs or of VMCs showing malignant transformation and also several cases of peripheral ICC, with histopathological similarities to VMCs (Figure 3(b)), arising from VMCs have been reported, raising the question of its potential role as an ICC precursor lesion [8, 27–29]. In such cases, VMCs were also found in nonneoplastic parts of the liver. For example, Xu et al. reported two cases of peripheral ICCs occurring in the liver with multiple VMCs. These cases suggested that VMCs may be a risk factor for the development of peripheral ICC, and malignant transformation has been reported in multiple VMCs [8].

In addition to the results obtained from human cases, a histological survey of hepatic parenchyma adjacent to ICC, as well as isolated regions of grossly normal livers, in an experimental animal model of ICC, which is characterized by a* K-ras* mutation and the deletion of p53, revealed several premalignant lesions [30]. Among them, some lesions, frequently found in animals with ICC, were similar to VMCs. ICCs appeared to arise directly from adjacent VMCs. The presence of these lesions in regions distal to the primary tumors suggests the multifocal initiation of VMC-like precursor lesions followed by the development of ICC. The findings of VMCs among mutant* K-ras-p53* animals provided experimental evidence for a progression model of ICC that includes VMCs.

### 2.2. Survey of Candidate Lesions in Our Cases

**Figure 4:**
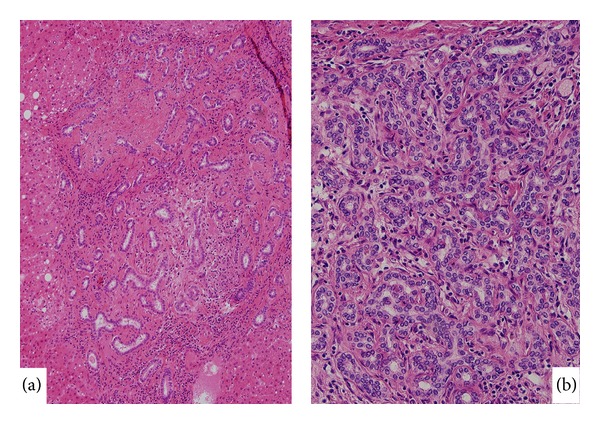
Bile duct adenoma-like lesion. (a) Small nodule composed of abundant interlobular bile ducts and fibrous stroma is found in the deep part of the liver. HE. (b) Small nodule of dense small interlobular bile ducts is found in the deep part of the liver. HE.

The above-mentioned, three biliary lesions were surveyed in our cases (Table 1). In these cases, chronic biliary diseases such as hepatolithiasis and primary sclerosing cholangitis were not found. As shown in Table 2, biliary adenofibroma was not found in the background liver or at the rim of peripheral CCs. As for typical, subcapsular BDA, it was found in one of 25 HCC cases and one of 18 cHC-CC cases, but not in peripheral ICC. Instead, bile duct adenoma-like lesions (Figures 4(a) and 4(b)) which were a little different from BDA, itself, were found in the background liver of one-third of peripheral ICCs (32.4%). These lesions were composed of dense and localized cluster of matured interlobular bile ducts, and their overall size was rather small ranging from 1 mm to 3 mm. They were alone or several in a given liver specimen and were found in fibrous septa or enlarged portal tract of the deep hepatic parenchyma. While these lesions were also found in 33.3% of cHC-CC cases, such lesions were infrequent in perihilar CC and HCC (11.8% and 12%, resp.), suggesting that this type of lesion could be related to peripheral ICC. As for VMCs, they were found focally and multiple in 24.3% of peripheral ICC cases and also other three controls (17.6% of hilar CC, 28% of HCC, and 33.3% of cHC-CC).

**Figure 5:**
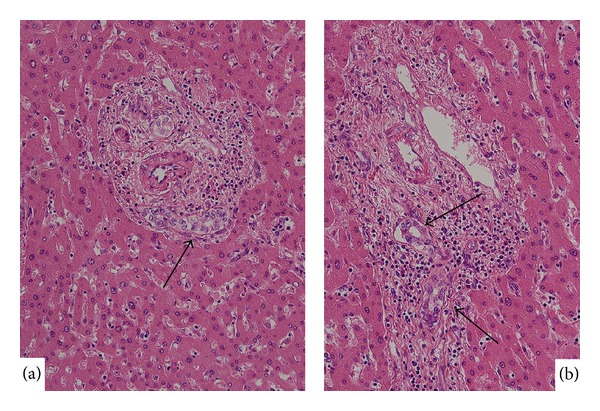
Atypical small bile duct lesion. (a) Small interlobular bile duct shows cellular and nuclear atypia. HE. (b) Small interlobular bile duct shows nuclear atypia and disturbed polarity. HE.

As a precursor or premalignant lesion of CC, BilINs are known and they are usually found in intrahepatic large bile ducts and perihilar and extrahepatic bile ducts [4, 5]. BilIN lesions were histologically classified as BilIN-1 (mild atypia), BilIN-2 (moderate atypia), and BilIN-3 (severe atypia including* in situ* carcinoma). While histological features of BilIN lesions were documented, it remains controversial whether BilIN-1 lesions contain some reactive hyperplastic changes. So, in this histological survey, only BilIN-2 and -3 lesions evaluated as neoplastic or preinvasive epithelial lesions were surveyed. In fact, it was found in this study that BilIN-2/3 lesions were found frequently in hilar bile ducts and peribiliary glands of hilar CC (52.9% and 50%, resp.) (Table 2). However, such lesions were infrequent or rare in these biliary anatomical components of biliary tree in peripheral CC, cHC-CC, and HCC. In our clinical experience, dysplastic biliary epithelial changes sharing features of BilIN are occasionally encountered in small bile ducts in peripheral CCs (Figures 5(a) and 5(b)). They showed that pleomorphic nuclei, nuclear hyperchromasia or stratification, and this size of affected bile ducts were enlarged, but not so atypical for making a diagnosis of CC or intraductal spread of carcinoma from CC. So, we surveyed such lesions in small bile ducts remote from CC itself of peripheral CC cases. It was found in this study that such small bile ducts showing atypical features were focally found in 10.8% of peripheral ICC. Interestingly, such lesions were found in 5.6% of cHCC-CC and not found in HCC and hilar CC. Further pathological and molecular studies are warranted for such small bile duct lesions which have not been reported in the literatures.

## 3. Progression from Low Grade Malignant or Borderline Biliary Lesions to More Aggressive ICC 

Peripheral ICC is characterized by an aggressive course and early metastasis with a poor prognosis [7]. However, the histological features of ICC are known to be heterogeneous, with low grade malignancies or borderline biliary epithelial lesions with blunt histologies being reported among them [20, 30]. The foci of conventional ICCs have also been observed within these lesions and such low malignant lesions remain at the rim of ordinary peripheral ICC [18, 20], suggesting the transition of these lesions to conventional ICC with a more aggressive lesion. The following examples have been reported.

### 3.1. Cholangiolocellular Carcinoma (Bile Ductular Carcinoma)

Cholangiolocellular carcinoma (CLC), a subtype of CC, exhibits the characteristic features of small monotonous and/or anastomosing glands. These tumors are accompanied by a variable fibrous stroma. Epithelial components are typically benign in appearance with blunt histologies showing the features of reactive bile ductules, and CLC is thought to originate from the bile ductules/canals of Hering, in which hepatic progenitor cells (HPCs) are located [13, 20]. These cells are also positive for hepatic progenitor markers, such as neural cell adhesion molecule (NCAM) in addition to CK7 and CK19.

While the majority of these tumors are mainly composed of neoplasms that appear to be benign, there are frequently foci of papillary and/or clear glandular formation with mucin production, representing CC areas. The latter lesion is commonly located in the central part of the tumor, while CLC components are typically found in the peripheral part. Transitional zones have also been reported in these tumors, suggesting that CLC is followed by ordinary peripheral ICC.

### 3.2. Cholangiocarcinoma with DPM Features

**Figure 6:**
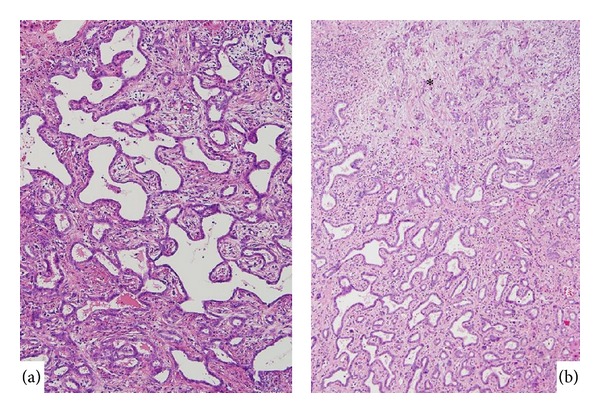
(a) Intrahepatic cholangiocarcinoma with features of ductal plate malformation. They appear to be blunt and lack aggressive features (low grade malignancy). (b) Within this type of cholangiocarcinoma (lower half), there are foci of ordinary cholangiocarcinoma (∗). HE.

We recently reported an atypical form of peripheral highly differentiated adenocarcinoma (peripheral ICC) mimicking DPM features [19]. The ductal plate (DP) can be found in certain developmental stages of the fetal liver, and DPM was used for an excess of embryonic bile duct structures with a DP configuration, reflecting the lack of remodeling in the DP [31, 32]. Microscopically, the tumor was composed of many vague, small nodular carcinomatous areas with desmoplastic reactions, and neoplastic glands had an irregularly dilated lumen lined with a single layer of cuboidal or low columnar carcinoma cells and irregular protrusions and bulges (Figure 6(a)), which resembled DPM [19]. At its border, the tumor appeared to replace nonneoplastic hepatic lobules or regenerative nodules. The central parts of the tumor were more or less hypocellular and fibrotic. The Ki-67 labeling index was less than 10% and the expression of p53 was very low. This subtype is thought to originate from bile ductules with DPM features. The foci of ordinary CC with aggressive histological features were observed in approximately half of these tumors [19], suggesting the transition of well-differentiated ICC with DPM features to more aggressive ordinary ICC (Figure 6(b)).

### 3.3. Rim of Low Grade or Borderline Malignancies in Conventional ICC

**Figure 7:**
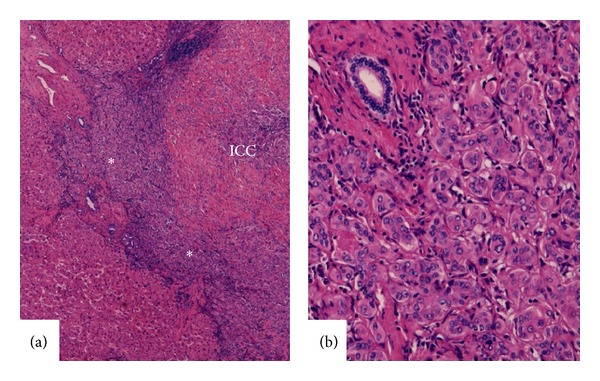
At the periphery (∗) of intrahepatic cholangiocarcinoma (ICC), there is well-differentiated lesion or bile duct adenoma-like lesion. (a) Lower magnification of ICC and (b) higher magnification of the rim (∗).

Peripheral ICC is typically well- to moderately differentiated tubular adenocarcinoma, while cord-like patterns are also frequently reported. While their histologies are commonly atypical enough to diagnose malignancy, highly differentiated adenocarcinoma or the appearance of borderline malignancy is occasionally observed at the peripheral rim of such ICCs, including the above-mentioned so-called benign lesions (bile duct adenoma, biliary adenofibroma, and VMCs) (Figures 7(a) and 7(b)), CLC (bile ductular carcinoma), and CC with DPM-like structures. These combinations suggest the progression of CLC or CC with DPM-like structures to more malignant or aggressive ICC. Based on the findings of our recent study [10], the foci of DPM-like features and foci of CLC were identified in approximately 10% and 30% of peripheral ICCs examined, respectively. These findings suggest that dedifferentiation may have occurred in preceding CLC or ICC with DPM-like structures and that the previous lesion remains as the peripheral rim.

**Figure 8:**
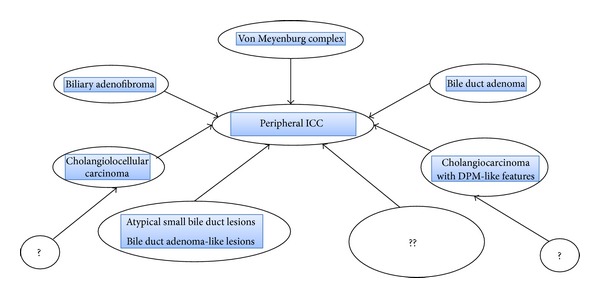
Schema of precursor lesions of peripheral intrahepatic cholangiocarcinoma (peripheral-ICC). Some of so-called benign lesions such as bile duct adenoma, von Meyenburg complex, and biliary adenofibroma are occasionally followed by the development of peripheral-ICC, and some of low grade cholangiocarcinoma (cholangiolocellular carcinoma and cholangiocarcinoma with ductal plate malformation (DPM)-like features) are also followed by ordinary peripheral-ICC. In addition, bile duct adenoma-like lesion and atypical small bile duct lesions described here could be also related to the development of peripheral ICC. Unidentified factors or lesions and may also be involved in the development of peripheral ICC.

In conclusion, several precursor or early neoplastic lesions of peripheral ICC reported in the literature were reviewed in the present study (Figure 8). Bile duct adenoma, biliary adenofibroma, and VMCs are reportedly associated with histologic features of malignancy or borderline lesions, and these lesions may be followed by carcinoma in a minority of peripheral ICC. Survey of unusual or atypical biliary lesions in peripheral ICC showed that bile duct adenoma-like lesions and atypical biliary lesions of small bile ducts were not infrequent in the background liver, possibly related to the development of peripheral ICC. In addition, several borderline or low grade biliary malignancies such as CLC or ICC with DPM-like structures frequently contain the foci of ordinary peripheral ICC, suggesting their transition to peripheral ICC and multistep cholangiocarcinogenesis. In hilar and extrahepatic CCs, the precursor lesions such as BilIN and IPN of bile duct are now being recognized and have been studied actively. More extensive surveys on precursor or premalignant lesions as well as more sophisticated studies based on pathologically confirmed precursor lesions in peripheral ICC as well as on those in hilar and extrahepatic CCs may lead to the earlier detection of and a better prognosis after surgical resection of peripheral ICC and hilar and extrahepatic CCs.

## Figures and Tables

**Table 1:** Main clinicopathologic features of the cases studied.

	Peripheral ICC	Perihilar CC	HCC	cHC-CC
Number of cases	37	34	25	18
Age (years)	65 ± 1.8	61 ± 1.7	62 ± 2.1	63 ± 1.8
Sex (male/female)*	21/16	19/15	17/8	14/4
Hepatitis virus*				
HBsAg (+)	6	1	9	6
HCV (+)	7	3	13	6
HBsAg (+)/HCV (+)	0	0	0	1
HBsAg (−)/HCV (−)	24	30	3	6
Cirrhosis*	4	1	25	11
Tumor size*				
Φ > 3 cm	31	20	10	8
Φ≦3 cm	6	11	15	9

*Number of cases. CC: cholangiocarcinoma; ICC: intrahepatic CC; HCC: hepatocellular carcinoma; cHC-CC: combined hepatocellular cholangiocarcinoma.

**Table 2:** Occurrence of biliary epithelial lesions related to cholangiocarcinoma.

	Peripheral ICC	Perihilar CC	HCC	cHC-CC
	(*n* = 37)	(*n* = 34)	(*n* = 25)	(*n* = 18)
Biliary fibroadenoma	0 (0%)	0 (0%)	0 (0%)	0 (0%)
Bile duct adenoma	0 (0%)	0 (0%)	1 (4%)	1 (5%)
Von Meyenburg complex	9 (24.3%)	6 (17.6%)	7 (28.0%)	6 (33.3%)
Bile duct adenoma-like lesion	12 (32.4%)	4 (11.8%)	3 (12%)	6 (33.3%)
Atypical small bile duct lesion	4 (10.8%)	0 (0%)	0 (0%)	1 (5.6%)
Biliary epithelial neoplasm (BilIN) 2/3				
Perihilar bile duct	6 (16.2%)	18 (52.9%)	1 (4%)	2 (11.1%)
Peribiliary glands	4 (10.8%)	17 (50.0%)	0 (0%)	1 (5.6%)

CC: cholangiocarcinoma; ICC: intrahepatic CC; HCC: hepatocellular carcinoma; cHC-CC: combined hepatocellular cholangiocarcinoma.
